# Using a National Representative Sample to Evaluate the Integrity of the 30-Day Surgical Mortality Metric

**DOI:** 10.1007/s10916-019-1288-3

**Published:** 2019-04-25

**Authors:** Yixian Qiu, Robert E. Freundlich, Sara Nelson, Catherine Clark, Jesse M. Ehrenfeld, Jonathan P. Wanderer

**Affiliations:** 1 Department of Emergency Medicine, Ohio State Wexler School of Medicine, Columbus, OH USA; 2 Department of Anesthesiology, Vanderbilt University Medical Center, Medical Arts Building 422 F, Nashville, TN 37212 USA; 3 Department of Biomedical Informatics, Vanderbilt University Medical Center, Nashville, TN USA; 4 Department of Surgery, Vanderbilt University Medical Center, Nashville, TN USA; 5 Department of Health Policy, Vanderbilt University Medical Center, Nashville, TN USA

**Keywords:** Total quality management, Palliative care, Hospital mortality

## Abstract

The 30-day surgical mortality metric is endorsed by the National Quality Forum for value-based purchasing purposes. However, its integrity has been questioned, as there is documented evidence of hospital manipulation of this measure, by way of inappropriate palliative care designation and changes in patient selection. To determine if there is evidence of potential manipulation, we retrospectively analyzed 1,725,291 surgical admissions from 158 United States hospitals participating in the National Inpatient Sample from 2010 to 2011. As a way of evaluating unnecessary life-prolonging measures, we determined that a significant increase in mortality rate after post-operative day 30 (day 31–35) would indicate manipulation. We compared the post-operative mortality rates for each hospital between Post-Operative Day 26–30 and Post-Operative Day 31–35 using Wilcoxon signed-rank tests. After application of the Bonferroni correction, the results showed that none of the hospitals had a statistically significant increase in mortality after post-operative day 30. This analysis fails to impugn the integrity of this measure, as we did not identify any evidence of potential manipulation of the 30-day surgical mortality metric.

## Introduction

Healthcare quality improvement has led to increased transparency, resulting in many performance metrics becoming available to the public as measurements of success in healthcare delivery. The publicity of these metrics has incentivized competition between hospitals and other healthcare organizations [1]. Structural measurements, process measurements and outcome measurements are often used when reporting the quality of surgical care. Examples include procedural volume, pre-operative venous-thromboembolic prophylaxis, and functional health status or mortality rate [2]. Thirty day surgical mortality is a metric that is widely used by the federal government, payers, and quality groups as a measure of postoperative risk and surgical success [3]. It was recently endorsed by the National Quality Forum (NQF) and may eventually be used for value-based purchasing, specifically following coronary artery bypass graft (CABG) surgery [4].

However, the integrity of the 30-day mortality metric has been called into question as there is potential for hospitals to participate in gaming – “distorting the process of care in order to meet targets or manipulating data to misrepresent actual performance” [5]. Additionally, there is evidence to support that manipulation of performance metrics compromises the quality of patient care. An example of this “cost-quality trade-off” is the scandal at Veterans Affairs (VA) hospitals where wait-time manipulation was implicated in the deaths of dozens of patients [6, 7]. A recent study performed by Hua et al. [8] examined whether public reporting of 30-day mortality delayed the decision to withdraw life-sustaining therapies in coronary artery bypass patients in Massachusetts and New York. The authors concluded that there was no evidence of increased mortality occurring immediately after day 30.

While Hua et al. did not find evidence in a narrow patient population, we sought to look for the affect more broadly. In this study, we sought to assess the extent to which the 30-day surgical mortality metric may be manipulated to artificially distort quality metrics in a national, multi-specialty patient sample. We analyzed a large national database comprised of the National Inpatient Sample (NIS) and National Surgical Quality Improvement Program (NSQIP), for evidence of prolonged life-sustaining care to improve 30-day surgical mortality. We based our analysis on the assumption that delaying initiation of palliative care or withdrawing of intensive care until after day 30 would improve the 30-day surgical mortality rate, [9–11] and hypothesized that if this metric were being artificially manipulated, hospitals would have a significant increase in mortality immediately after Post-Operative Day (POD) 30.

## Methods

This study was approved by the Vanderbilt University Medical Center Institutional Review Board (#161027) and the follows the Strengthening the Reporting of Observational Studies in Epidemiology (STROBE) checklist [12].

### Process of hospital selection, characteristics assessment

The NIS is a large, publicly-available, all-payer, inpatient care database of the United States, compiled by the Agency for Healthcare Research and Quality. Federal government-owned hospitals are not represented in the NIS database [13]. We analyzed hospitals participating in the sample from 2010 to 2011. To select a cohort from this extensive database and optimize the signal-to-noise ratio of our analysis, we included only those hospitals who had at least 100 surgical patients who had been hospitalized ≥30 days. Statistical analyses were performed using R version 3.3.2 (R Foundation for Statistical Computing, Vienna, Austria).

### Statistical analysis

Descriptive statistics for each hospital including ownership, location, teaching status and regions are represented as counts and percentages. To visually analyze the mortality trends around POD 30, we plotted Kaplan-Meier survival curves up to POD 60 for each hospital individually, as well as all hospitals combined. We then calculated the daily mortality rate from POD 26 to POD 35 for each institution and calculated the mortality rates between POD 26–30 and POD 31–35 for each institution using the Wilcoxon Rank Sum Tests with a Bonferroni correction, to adjust for multiple comparisons.

## Results

### Selected hospitals and their characteristics

A total of 1,725,291 surgical cases from 158 hospitals were included for analysis. The hospitals’ demographic information is presented in Table 1. Most hospital ownership was private, non-profit (66.5%), followed by government, nonfederal (18.4%) and private, invest-own (10.8%). Most of the hospitals analyzed were considered large (73.4%), defined as greater than 100 beds for rural; 200 beds for urban non-teaching; and 500 beds for urban teaching. Most hospitals represented were urban (93.7%) and were teaching hospitals (77.2%). All regions of the country were represented with 23.4% from the Northeast, 13.9% from the Midwest, 40.5% from the South and 22.2% from the West. Among the cases analyzed, approximately half of the cases were elective/scheduled admissions (47.7%), while the remaining were non-elective/emergent.

**Table 1:** Hospitals’ Characteristics

Characteristic	N (%) (*N* = 158)
Ownership:	
Government, nonfederal	29 (18.4%)
Private, non-profit	105 (66.5%)
Private, invest-own	17 (10.8%)
Unknown	7 (4.4%)
Size:	
Small	9 (5.7%)
Medium	26 (16.5%)
Large	116 (73.4%)
Unknown	7 (4.4%)
Location:	
Urban	148 (93.7%)
Rural	3 (1.9%)
Unknown	7 (4.4%)
Region:	
Northeast	37 (23.4%)
Midwest	22 (13.9%)
South	64 (40.5%)
West	35 (22.2%)
Teaching Status:	
Teaching	122 (77.2%)
Non-teaching	29 (18.4%)
Unknown	7 (4.4%)
Case Risk Level:	
Elective	47.70%
Non-elective	52.10%
Unknown	0.20%

### 30-Day mortality results of selected hospitals

**Fig. 1:**
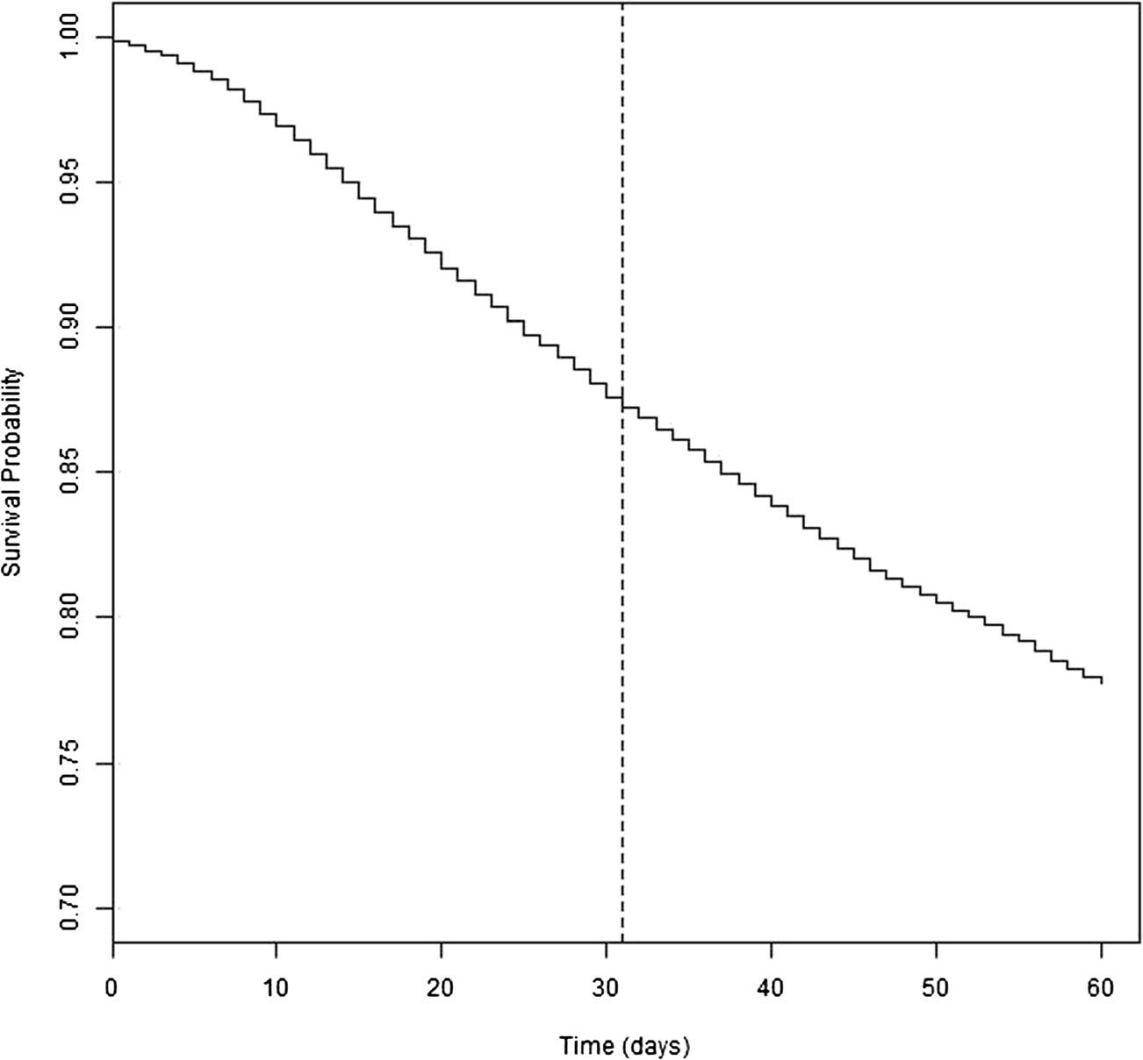
All-Hospital Kaplan Meier Survival Curve for Post-Operative Day 0–60

Figure 1 shows the Kaplan Meier survival curve for patients in all hospitals combined (*n* = 1,725,291). Visual inspection of the curve shows there was no dramatic increase in mortality rate (or drop in the survival curve) around POD 30. This finding was supported by the results of the Wilcoxon Rank Sum Tests; across all 158 hospitals analyzed, no hospital had a significant *p* value after application of the Bonferroni correction (significance: *p* ≤ 0.00029). This indicates that the mortality rate did not change in POD 31–35 period.

## Discussion

### Implications of the analysis

Thirty-day surgical mortality is used increasingly by buyers and payers to assess postoperative risk and surgical success and is endorsed by the NQF for value-based purchasing—specifically for CABG surgery. [4] In this analysis performed on a large, national patient sample, our results showed that of the 158 institutions that had at least 100 patients with a length of stay greater than or equal to 30 days, there was no evidence of manipulation of the 30-day surgical mortality metric. These results support the integrity of the metric and are consistent with prior research [8].

### Evidence of quality metric gaming

A performance-based healthcare setting creates incentives for manipulating quality metric data, even though the quality measures ostensibly exist to improve healthcare outcomes [14]. Multiple authors have discussed incidents of such “gaming”. A compelling example is provided by the experience of cardiac surgeons in New York. The public release of individual surgeons’ mortality performance by the New York State Department of Health has led to denial of surgical treatment for high-risk patients [15]. Another instance of abuse of performance data is the drastic increase in the percentage of deaths recorded under palliative care in United Kingdom (UK) from 2006 to 2013. This increase points to hospitals’ adjustment to the ratio of expected deaths to number of patients admitted into palliative care [5, 16]. Additionally, a sensitivity analysis conducted in the UK, which focused on the Hospital Standardized Mortality Ratio (including one sub-analysis based on 30-day total mortality), found that the metric correlates poorly to the proportion of deaths within 30 days of hospital readmission [17].

In a discussion on the introduction of performance league tables (data that highlights surgeons’ performance) for UK surgeons and hospitals, the British Medical Journal reported that hospitals can simply manipulate the data by transferring patients, changing the operative class, refusing to operate or selecting the most profitable patients for elective procedures [18]. Similarly, Chatterjee et al. reported that cardiology quality measures have been maneuvered with upcoding (coding a patient in a way to make them appear sicker), reclassifying a patient’s diagnosis, and excluding the case from quality metrics [19].

This type of evidence of system manipulation proved that system gaming is present and should be continually monitored. However, our results, and the results presented by Hua et al. [15], did not reveal any instance of hospitals prolonging life-sustaining measures in order to manipulate 30-day surgical mortality metrics, supporting the reliability of this metric. However, it is essential to continue asking the following questions: “Is 30-day surgical mortality a good metric for surgical success?” and “How do we veer hospitals away from manipulating this metric?” Birkmeyer et al. explored the potential advantages and disadvantages of outcome-based quality metrics such as 30-day surgical mortality [2]. We agree with their assessment that such measurements alone may result in better surgical performance, but these metrics may be inadequate and imprecise, especially for hospitals with low caseloads as this is usually indicative of low case risks [2]. In contrast, such measurements are more desirable for hospitals with higher caseloads conducting procedures of greater baseline risk: for example, urban tertiary care centers with a large cardiothoracic subdivision that performs many CABG procedures [2].

### Strengths and limitations

Strengths of our study include use of a large and representative sample, as well as a flexible methodology that can be easily applied to other hospitals of any size or in other databases. Furthermore, we have a large base of evidence from examples in medical literature. Using one means of analysis, we did not show any evidence of the metric being manipulated; however, the strategic employment of data could have gone undetected in this sample due to factors other than the lack of its existence. For example, for-profit private institutions account for 6% of our sample, and one can argue that the incentives for these institutions may make them more prone to data manipulation. We should retain a healthy skepticism and remain aware of the possibility for manipulation that we have failed to recognize, consistent with some of the high-profile examples we have cited.

Our selection process for the hospitals focused on those with at least 100 patients staying past 30 days during our study period. This inevitably restricts the representation of the analyzed hospitals to predominantly larger, tertiary institutions in urban areas. Furthermore, due to the nature of the NIS database, the mortality in this study is limited to inpatient deaths and does not include outpatient or nursing home deaths, as well as mortality after transfer to another healthcare facility. However, the study focused on the evidence of prolonging life via life-sustaining measures in the index hospitalization.

Additionally, although the NIS database provided organ-based problem lists and the ICD-9 codes for diagnoses for each patient, the surgical case types were not specified (i.e. cardiothoracic versus trauma versus neurological). An analysis of data sources that incorporate case-mix index could offer more insight on whether specific specialties contribute to manipulation of such metrics.

### Future research directions

We have provided a methodology to assess whether potential gaming exists for hospitals of any size. Future application of our methodology should include more institutions that are for-profit and those from a more recent database. If a statistically significant change of mortality is observed, future studies should analyze mortality change by year and compare the mortality to that of the same hospitals whose data is found in other databases.

The NIS database, as previously described, did not include the specific types of cases that contribute to the surgical mortality for each hospital analyzed. Future analysis using a database with more data on surgical case-mix, such as NSQIP, will answer whether there is evidence of metric gaming within specific specialties or departments. [20]

Finally, since multiple resources have reported suspicions or cases where hospitals delay necessary palliative care until after Post-Operative Day 30, more specific data showing number of days before acquisition of necessary should be explored.

## Conclusion

Our analysis of a sample of United States hospitals from a national database found that among those examined, none had a significant increase in mortality after Post-Operative Day 30. This suggests there was no manipulation of the 30-day surgical mortality metric, supporting the integrity of the measure.
